# Size-Dependent Biodistribution of Fluorescent Furano-Allocolchicinoid-Chitosan Formulations in Mice

**DOI:** 10.3390/polym13132045

**Published:** 2021-06-22

**Authors:** Iuliia Gracheva, Maria Konovalova, Dmitrii Aronov, Ekaterina Moiseeva, Alexey Fedorov, Elena Svirshchevskaya

**Affiliations:** 1 Department of Organic Chemistry, Nizhni Novgorod State University, Gagarina av. 23, 603950 Nizhni Novgorod, Russia; afedorovnn@yandex.ru; 2 Shemyakin-Ovchinnikov Institute of Bioorganic Chemistry RAS, Miklukho-Maklaya St. 16/10, 117997 Moscow, Russia; mariya.v.konovalova@gmail.com (M.K.); aronov.mml@gmail.com (D.A.); evmoise@gmail.com (E.M.); esvir@yandex.ru (E.S.)

**Keywords:** furano-allocolchicinoid, chitosan, nano/micro particles, biodistribution, Wnt-1 breast tumor

## Abstract

The aim of this study was to compare the biodistribution in mice of functionalized rhodamine B (Rh) labeled colchicine derivative furano-allocolchicinoid (**AC**, **6**) either conjugated to 40 kDa chitosan (**AC-Chi**, **8**) or encapsulated into chitosan nanoparticles (**AC-NPs**). AC-NPs were formed by ionotropic gelation and were 400–450 nm in diameter as estimated in mice by dynamic light scattering and confocal microscopy. AC-Chi and AC-NPs preserved the specific colchicine activity in vitro. AC preparations were once IV injected into C75BL/6 mice; muscles, spleen, kidney, liver, lungs, blood cells and serum were collected at 30 min, 2, 5, 10, and 20 h post injection. To analyze the distribution of the furano-allocolchicinoid preparations in body liquids and tissues, Rh was measured directly in sera or extracted by acidic ethanol from tissue homogenates. Preliminary Rh extraction rate was estimated in vitro in tissue homogenates and was around 25–30% from total quantity added. After in vivo injection, AC-NPs were accumulated more in liver and spleen, while less in kidney and lungs in comparison with free AC and AC-Chi. Therefore, incorporation of colchicine derivatives as well as other hydrophobic substances into nano/micro sized carriers may help redistribute the drug to different organs and, possibly, improve antitumor accumulation.

## 1. Introduction

**Figure 1:**
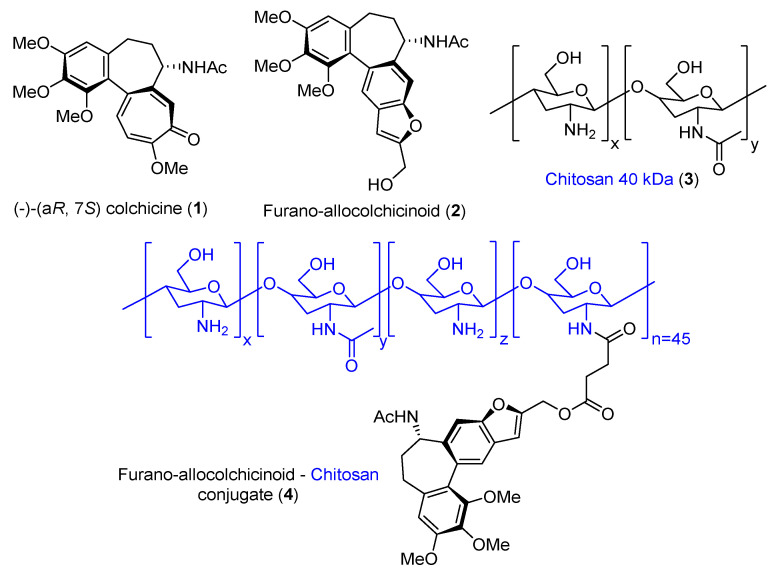
Structures of colchicine (**1**), furano-allocolchicinoid (**2**), chitosan 40 kDa (**3**) and furano-allocolchicinoid—chitosan conjugate (**4**) [7].

Natural alkaloid colchicine (**1**) (Figure 1) is a mitotic poison that binds to intracellular protein tubulin and prevents mitotic spindle formation. This leads to a block in cell proliferation and reduced cell motility [1,2]. Orally administrated colchicine has an elimination half-life of 20–40 h [3]. It binds to neutrophils and serum albumin and may cause the body to produce fewer blood cells of different types [4]. Colchicine is metabolized by intestinal and hepatic cytochrome CYP3A4 and excreted via the hepatobiliary and renal routes. The alkaloid significantly affects the gastrointestinal tract inducing nausea, vomiting, and diarrhea in 5–10% of patients even at approved doses [5], which prevents its usage in cancer treatment and other diseases. The low therapeutic index of colchicine, like many other tubulin-binging agents, is caused by its inability to yield concentrations high enough at the target site to trigger apoptosis, associated with the non-specific cytotoxicity towards normal tissues and organs. Numerous attempts have been made to modify the molecule in order to reduce its inherent toxic side effects [6].

Colchicine biodistribution studies demonstrated its bioaccumulation in liver, intestine, kidney, and heart at organ/muscle ratios 11, 5, 7, and 6, respectively [8]. Unspecific tissue partition of colchicine may be reduced by decorating it with hydrophilic groups such as polyethylene glycol (PEG) [9,10]. Another way to sequester colchicine from non-target organs is to incorporate it into a drug carrier which can improve the therapeutic antitumor index via “enhanced permeability and retention effect” [11] due to an increased uptake of macromolecules by tumors. 

Among various polymers used to develop drug carriers, chitosan (**3**, Figure 1) has significant advantages over analogues due to its biodegradability, low toxicity, and multiple amino groups used to obtain derivatives with desired properties [12]. Chitosan was used to develop various delivery systems for insulin, morphine, DNA, siRNA, proteins and peptides [13,14,15]. Chitosan based nanoparticles (NPs) can be developed as theranostic agents for both drug delivery and diagnostics [16,17,18]. 

The pharmacokinetics of nanosized carriers in living organisms is quite complex and depends on a number of factors, e.g., physical and chemical properties of NPs, surface functionalization, and permeability of various tissue membranes [19]. For the biodistribution analysis, it is essential to incorporate a reliable label into a drug or a delivery vehicle to monitor its accumulation in tissues and body fluids. In many cases experimental data are obtained by different poorly compatible methods, some of which have low sensitivity; and many studies provide limited information on ADME (absorption, distribution, metabolism, and excretion). For example, after investigation of biodistribution and pharmacokinetics of ^125^I-labeled PLGA NPs in mice, Panagi et al. [20] found that the accumulation of NPs mostly occurs in liver with minor amounts in lungs, intestine, and muscles, while kidney and gall inputs were not estimated. Flaten et al. [21] studied the biodistribution of ^3^H-labeled camptothecin and its liposome formulation in HT-29 mouse tumor model. Although all major organs were collected and studied, the analysis was performed 20 h post injection which made it impossible to analyze primary biodistribution and excretion. Body distribution of chitosan NPs loaded with a fluorescent dye and siRNA/cisplatin was studied using bioimaging for qualitative and PCR method for quantitative analysis in lung cancer nude mice model. Analysis was conducted from 12 to 120 h and demonstrated blood clearance and a major accumulation of NPs in liver, kidneys, and tumor [22].

Recently, our group synthesized a number of heterocyclic allocolchicinoids [23,24,25] possessing similar to colchicine antitumor activity but lower systemic toxicity. It was found that the conjugate (**4**) of colchicinoid **2** with chitosan **3** (40 kDa) (Figure 1) is more effective in tumor growth inhibition in mice compared to intact colchicinoid **2**, which was associated with a better entry of nanoparticles into tumor tissues and less systemic toxicity [7]. The goal of this work was to synthesize fluorescent furano-allocolchicinoid derivative (**AC**, **6**), fluorescent furano-allocolchicinoid—chitosan conjugate (**AC-Chi**, **8**) and to develop AC-chitosan nanoparticles (**AC-NPs**), and analyze their biodistribution in mice using fluorescent signal in tissues and body liquids as a quantitative label.

## 2. Materials and Methods

### 2.1. Materials

Commercially available reagents («Sigma-Aldrich», Steinheim, Germany; «Alfa Aesar», Kandel, Germany; «ACROS ORGANICS», Geel, Belgium) were used without additional purification. Column chromatography was performed using Macherey–Nagel Kieselgel 60 (70–230 mesh). ^1^H and ^13^C NMR spectra of **6** were recorded at room temperature in CD_3_OD on Agilent DD2 400 instruments. Chemical shifts (δ) are reported in parts per million (ppm) from tetramethylsilane (TMS) using the residual solvent resonance (CD_3_OD: 3.31 ppm for ^1^H NMR, 49.00 ppm for ^13^C NMR). Multiplicities are abbreviated as follows: s = singlet, d = doublet, t = triplet, q = quartet, m = multiplet; dd = doublet of doublets; dt = doublet of triplets; td = triplet of doublets). ^1^H-NMR spectra of the initial chitosan and chitosan derivatives were obtained on a Bruker DRX 500 spectrometer in 0.01 M DCl/D_2_O at 30 °C. EI mass spectra (70 eV) were obtained on a DSQ II mass-spectrometer (Thermo Electron Corporation, Austin, TX, USA) with a quadrupole mass-analyzer. MALDI mass spectra were obtained on a MALDI-TOF mass-spectrometer Bruker Microflex LT.

Medium molecular weight (MW) chitosan (≈40 kDa) with deacetylation degree 0.94 (Aladdin Chemistry Co., Ltd., Shanghai, China); Chitosan with MW ≈ 200 kDa and deacetylation degree 0.85 was purchased from ZAO «Bioprogress» (Moscow, Russia). Samples were purified by sequential precipitation with 30% CH_3_COOH and 12% NH_4_OH. Caproic anhydride (Fluka, Germany), sodium tripolyphosphate (Sigma-Aldrich, St. Louis, MO, USA), calcium chloride (Pacreac, Barcelona, Spain), acetic acid, ammonium hydroxide (Chimmed, Moscow, Russia), ethylenediaminetetraacetic acid (EDTA), (Sigma-Aldrich, St. Louis, MO, USA), Dulbecco’s modified Eagle’s medium (DMEM), RPMI 1640, phosphate-buffered saline (PBS), *L*-glutamine, ampicillin, Rodamine B (Sigma-Aldrich, St. Louis, MO, USA) were used as received.

### 2.2. Synthesis of Fluorescently Labeled Furano-Allocolchicinoid (**6**)

**Scheme 1:**
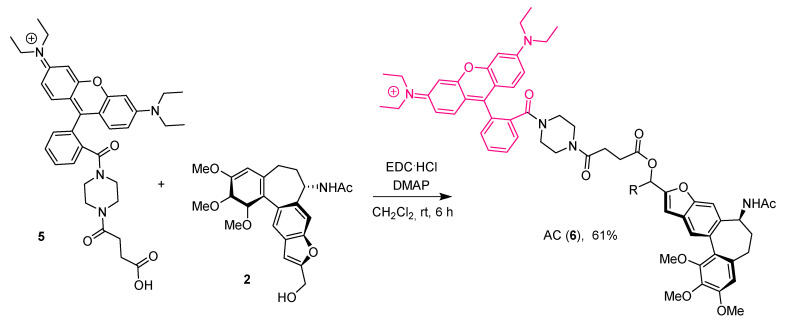
Structures and synthesis of rhodamine B-labeled furano-allocolchicinoid (**6**).

Compound **2** was prepared according to the previously proposed procedure from commercial colchicine [23]. Compound **5** (see Scheme 1) was synthesized from commercial rhodamine B according to the method proposed by Nguyen and Francis [26]. 

A Schlenk flask was degassed, filled with argon, and charged with EDC·HCl (28 mg, 0.146 mmol) and compound **5** (45 mg, 0.073 mmol). Anhydrous CH_2_Cl_2_ (0.5 mL) was added, and the mixture was stirred for 30 min at 0 °C. A second Schlenk flask was charged with furano-allocolchicine **2** (30 mg, 0.073 mmol) and DMAP (5 mg, 0.036 mmol). The flask was then filled with argon and dry CH_2_Cl_2_ (0.5 mL) was added. The mixture from the first flask was transferred into the second flask by using a syringe. The resulting mixture was stirred at 0 °C for 1 h and then at room temperature for 3 h. After the solvent removal under reduced pressure the residue was dissolved in H_2_O (30 mL), saturated with NaCl and then extracted with multiple portions of 2:1 *i*PrOH/CH_2_Cl_2_ until a faint pink color persisted. The combined organic layers were dried over Na_2_SO_4_ and concentrated under reduced pressure. The product was purified by column chromatography, eluent: petroleum ether/ethyl acetate/ethanol (1:1:1) to give **6** as a dark purple solid (45 mg, 0.045 mmol, 61%). ^1^H NMR (400 MHz, CD_3_OD) δ 8.09 (d, *J* = 6.6 Hz, 2H), 7.76 (d, *J* = 5.9 Hz, 2H), 7.68–7.65 (m, 1H), 7.59 (s, 1H), 7.53–7.50 (m, 1H), 7.43 (s, 1H), 7.28 (d, *J* = 9.5 Hz, 2H), 7.07 (d, *J* = 8.7 Hz, 2H), 6.95 (s, 2H), 6.82 (s, 1H), 6.75 (s, 1H), 5.22 (s, 2H), 4.74 (dd, *J* = 11.9, 6.8 Hz, 1H), 3.89 (d, *J* = 8.2 Hz, 6H), 3.70–3.66 (m, 8H), 3.46 (s, 3H), 3.40 (s, 8H), 2.66 (s, 3H), 2.53 (dd, *J* = 12.7, 5.8 Hz, 2H), 2.25 (m, 4H), 2.03 (s, 3H), 1.29 (t, *J* = 6.8 Hz, 12H). ^13^C NMR (101 MHz, CD_3_OD) δ 173.91, 172.47, 159.28, 159.27, 157.21, 157.01, 156.21, 154.16, 153.81, 152.14, 143.03, 142.44, 139.19, 136.60, 133.18, 132.27, 131.76, 131.29, 130.88, 128.94, 127.76, 126.38, 123.82, 115.42, 114.87, 109.05, 108.11, 107.77, 106.55, 97.36, 61.62, 61.41, 59.66, 56.62, 50.80, 46.91, 39.87, 39.78, 31.42, 30.68, 30.08, 28.63, 22.68, 12.84. MS (MALDI-TOF) = 1003.9 [M]^+^. Preparation **6** is designated as **AC** in the text.

### 2.3. Synthesis of Fluorescently Labeled Furano-Allocolchicinoid-Chitosan Conjugate (**8**)

Rhodamine B derivative **5** was conjugated with **4** according to the published procedure [7]. Furanoallocolchicinoid-chitosan conjugate **4** (≈40 kDa, 50 mg, 0.001 mmol) was dissolved in 6 mL of distilled water, acidified with acetic acid to pH = 6 and diluted with 15 mL of methanol, after which **5** (23 mg, 0.037 mmol), EDC·HCl (28 mg, 0.148 mmol) and NHS (17 mg, 0.148 mmol) were added and the mixture was stirred for 24 h at room temperature. The resulting solution was dried under reduced pressure, washed with toluene, CH_2_Cl_2_ (5 × 50 mL) and dried in vacuo. The product **8** was obtained as a bright-pink solid mass (69 mg). For the in vivo experiments 10 mg of product **8** was dissolved in acidic 50% ethanol overnight at stirring. Final concentration of furano-allocolchicinoid was estimated by MTT assay using free furano-allocolchicinoid as a reference and was 0.7 mM [7]. Preparation **8** is designated as **AC-Chi** in the text.

### 2.4. Synthesis of N-Laurylchitosan (Kashirina et al., 2018)

Modification of chitosan with lauric and succinic residues is necessary for the reduction of the charge of preparations as negative *ζ*-potential of the drug used for IV injections is preferential [7]. Chitosan (Heppe Medical Chitosan GmbH, MW ≈ 40 kDa, deacetylation degree 0.86) 100 mg, was dissolved in 10 mL of 2% acetic acid and 30 mL of methanol, a solution of *N*-hydroxysuccinimide ether of lauric acid with different molar ratios (0.1–1 mmol) in 10 mL of methanol was added, and the reaction mixture was stirred for 12 h at rt. Methanol was evaporated, the aqueous solution was dialyzed against 0.1% acetic acid and freeze-dried. *N*-laurylchitosan was obtained in the form of a light white powder with 85% yield [27].

### 2.5. Synthesis of N-Laurylsuccinoylchitosan (Kashirina et al., 2018)

Succinic anhydride (50–500 mmol) was added to the *N*-laurylchitosan solution (80 mg, 2 mmol) in 8 mL of 2% acetic acid and 32 mL of methanol, and the reaction mixture was stirred for 12 h at rt. Methanol was evaporated, the aqueous solution was dialyzed against 0.1% acetic acid and freeze-dried. *N*-laurylsuccinoylchitosan was obtained in the form of a white powder (65 mg, 81%). ^1^H NMR (500 MHz, DCl/D_2_O) δ 2.04 (*N*-acetyl), 2.54 ((CH_2_)_2_, succinoyl); 1.21 (CH_2_, lauryl). The degree of substitution (succinoyl/lauryl) was calculated from the ratio of the integral intensity of the *N*-succinoyl- and *N*-lauryl-radical proton signals to the proton signal at 2C (δ 4.56). ^1^H NMR spectra are shown in Figure S1.

### 2.6. Preparation of N-laurylsuccinoylchitosan Nanoparticles Containing Furano-Allocolchicinoid 

Product **6** dissolved in DMSO (20 mM) was added drop-wise (0.02%) to *N*-laurylsuccinoylchitosan 30% alcohol solution (1 mg, 0.17 mmol) under magnetic stirring at 30 rpm until opalescence occurred, which was estimated by a Specol 11 spectrophotometer (Carl Zeiss Jena, Jena, Germany) at 590 nm. The pH of the reaction mixture was maintaining 7.4 with a 5M solution of Na_2_CO_3_. The reaction mixture was stirred for 1 h at rt. The solution was dialyzed against saline overnight. NPs were formed during dialysis. The particle diameter was determined by dynamic light scattering (DLS). *N*-Laurysuccinoylchitosan nanoparticles containing furano-allocolchicinoid (**6**) are designated as **AC-NPs** in the text.

### 2.7. Standardization of AC Formulations 

All furano-allocolchicinoid preparations contained rhodamine B bound to furano-allocolchicinoid. MFI was used as the main label in biodistribution studies. To obtain a comparable MFI injected, formulations were dissolved in saline or acidic ethanol to calibrate the quantity of MFI/mL. 

### 2.8. Cell Cultures

Human pancreatic PANC-1, ovarian HeLa tumor cell lines, immortalized embryonic kidney HEK293 cells and murine colon CT26 cell line were used in the study. HEK293 were grown in DMEM supplemented with 10% fetal calf serum (FCS), pen-strep-glut (all from PanEco, Moscow, Russia). All other cell lines were grown in RPMI-1640 with the same supplements. Cells were passaged by trypsinization using Trypsin/EDTA solution (PanEco, Moscow, Russia) twice a week. Twenty-four hours before the assays, cells were seeded in 96 well plates at 10^4^ cells/well and incubated overnight to achieve standardized growth conditions. 

### 2.9. MTT-Assay

**Figure 2:**
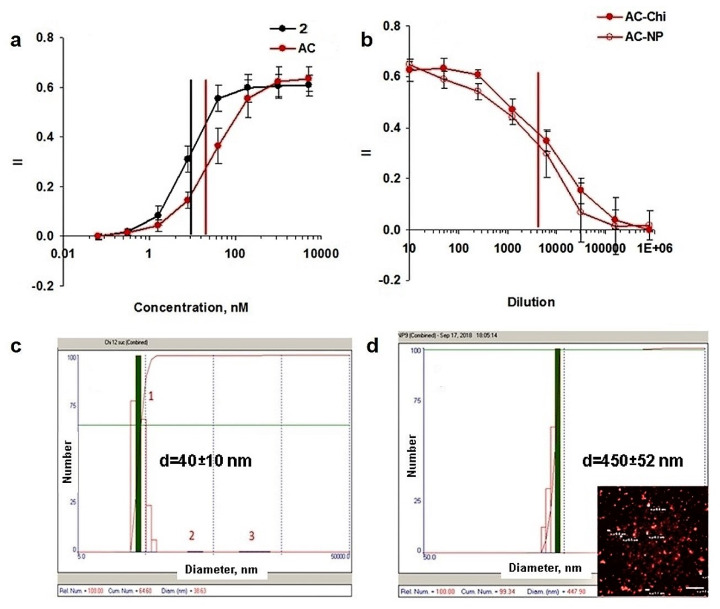
Cytotoxic activity of furano-allocolchicinoid **2** in different formulations. (**a**,**b**) Inhibition of cell proliferation by unlabeled (**2**, black) or rhodamine B labeled furano-allocolchicinoid (**AC**, red) (**a**); **AC-Chi** and **AC-NPs** (**b**) estimated by 72 h MTT assay in PANC-1 cells. IC_50_ is shown with vertical lines of corresponding colors. (**c**,**d**) Average diameter of **AC-Chi** (**c**) and **AC-NPs** (**d**) estimated by dynamic light scattering. Insertion shows **AC-NPs** confocal image. Bars show diameters of some nanoparticles.

Cytotoxic effect of different formulations was estimated by a standard 3-(4,5-dimethyl-2-thiazolyl)-2, 5-diphenyl-2*H*-tetrazolium bromide (MTT, Sigma, St. Louis, MO, USA) test as described earlier [28]. In short, different dilutions of the formulations using series of dilutions were prepared on a separate plate and then transferred in 100 µL to the plates with the cells. Non-treated cells served as controls. Plates were incubated for 72 h. For the last 6 h 250 μg/mL of MTT was added in 10 μL/well. After this, the incubation culture medium was removed and 100 μL of DMSO was added to each well. Plates were incubated at shaking for 15 min to dissolve formazan. Optical density was read on spectrophotometer Titertek (UK) at 540 nm. Results were analyzed by Excel package (Microsoft). Cytotoxic concentration giving 50% of dead cells (IC_50_) varied between cells lines and is shown on Figure 2. The inhibition of proliferation (inhibition index, II) was calculated as [1 − (OD_experiment_/OD_control_)], where OD was MTT optical density.

### 2.10. Dynamic Light Scattering (DLS)

The average liposome and HC NP diameters were determined using 90 Plus Particle Size Analyzer (Brookhaven, NY, USA) in water (25.0 ± 0.1 °C) at a scattering angle of 90° and wavelength of 661 nm using Big Particle Sizing Software. Zeta potential of NPs was determined in 10 mM KCl solution using identical Big Pal Zeta-Potential analyzer hard-ware and software. 

### 2.11. Confocal Microscopy

AC-NPs were overloaded onto a microscopic glass slide, air dried, covered with a cover glass in a polymerizing medium Mowiol 4.88 (Calbiochem), and analyzed using Eclipse TE2000 confocal microscope (Nikon).

For the analysis of intestinal pathology fractions of small intestine were excised 5 h post injection of the furano-allocolchicinoid preparations, washed out from feces, fixed with 4% paraformaldehyde, frozen in Tissue-Tek (Sakura, Alphen aan den Rijn, The Netherlands), and cryosectioned (ThermoScientific, Waltham, MA, USA). Control sections were stained with DAPI to visualize nuclei; experimental sections were additionally stained with phallaidin-Alexa488 (Life technologies, Waltham, MA, USA) staining actin microfilaments. Analysis was conducted using confocal microscope Nikon E2000 (Tokyo, Japan). 

### 2.12. Flow Cytometry

To study leukocyte binding of furano-allocolchicinoid formulations were mixed with 1 mL of fresh mice blood, incubated 1 h at 37 °C in 5% CO_2_ conditions. Red blood cells were removed by lysing solution (BD, Franklin Lakes, NJ, USA). Samples were analyzed using gating to macrophages, neutrophils and lymphocytes on FACSCalibur device (BD, Franklin Lakes, NJ, USA). Total 20,000 events were collected. The results were analyzed using WinMDI 2.8 software. Total mean fluorescence intensity was summarized and percentages of fluorescence were calculated for each cell population. 

### 2.13. Extraction Efficiency

To estimate extraction efficacy in in vivo experiments 1 mL of tissue homogenates of liver, kidney, spleen or blood obtained from intact mice were prepared in saline. Equal fixed amount (20 μL) of fluorescent AC formulations were added to the homogenate samples and incubated overnight at +4 °C at shaking. After that 1 mL of 0.3N HCl in 70% ethanol extraction buffer [29] was added to each sample and again incubated overnight at +4 °C at shaking. The mixtures were then transferred to Eppendorf microcentrifuge tubes and centrifuged at 10,000 rpm for 20 min. Samples (200 µL) in triplets were transferred to black plates and the fluorescence was measured at 490 nm using Glomax Multi spectrofluorimeter (Promega, Madison, WI, USA). Control amount of furano-allocolchicinoid preparations were diluted in the same manner. Extraction efficacy was calculated using the following equation: EE = (mean fluorescence intensity (MFI) in samples)/(MFI in controls)·100. 

### 2.14. Biodistribution Experiments

#### 2.14.1. Mice 

C57BL/6 mice were purchased from Pushchino Affiliation of Shemyakin-Ovchinnikov Institute of Bioorganic Chemistry RAS, Moscow. All mice were 6–8 weeks old and maintained in minimal pathogen animal facility at the Shemyakin-Ovchinnikov Institute of Bioorganic Chemistry RAS, Moscow. 

Mice were allocated in three groups and were injected IV in the tail vein with rhodamine labeled AC, AC-Chi, or AC-NPs. Experiments were repeated 4 times and pooled results are presented. Totally 12 mice per time point were used in each group.

#### 2.14.2. Ethical Approval

All studies were conducted in an AAALAC accredited facility in compliance with the PHS Guidelines for the Care and Use of Animals in Research, protocol #232 from 24 May 2018.

#### 2.14.3. Rhodamine Extraction from Organs

At different time intervals blood was collected from the orbital sinus of anaesthetized (Isoflurane, Baxter Healthcare, San Juan, Puerto-Rico) mice and coagulated. Serum was used directly without extraction. Mice were sacrificed by cervical dislocation at different times post injection (0.5, 2, 5, 10, 20 h). Residual blood in organs was removed by transcardial perfusion by heparinized saline solution. Thigh muscle piece, spleen, kidneys, liver, and lungs were collected, weighed, and homogenized using steel strainer in 1 mL saline solution per 0.3 g of tissue weight. Rhodamine extraction from different tissues was conducted by acidic ethanol buffer [29]. To this end 1 mL of tissue homogenate was mixed with 1 mL 0.3N HCl in 70% ethanol and incubated overnight at +4 °C at shaking. The mixtures were then transferred to Eppendorf microcentrifuge tubes and centrifuged at 10,000 rpm for 20 min. Cleared samples (200 µL) in triplets were transferred to black plates and the fluorescence was measured at 490 nm using Glomax Multi spectrofluorimeter (Promega). Organs and serum from intact mice (*n* = 4) were used to estimate a cut-off limit of autofluorescence. The cut-off limit was subtracted from the mean fluorescence intensity (MFI) measured in 200 µL of cleared extracts. Biodistribution was analyzed among serum, muscles, leukocytes, spleen, kidney, liver, and lungs. In some experiments leukocyte binding was also included in the analysis. For this, blood was collected in heparinized tubes; red blood cells were lysed, and rhodamine form leukocytes was extracted as described.

#### 2.14.4. Statistical Analysis

Statistical analysis was performed using Excel software and Student’s *t*-test. Comparison values of *p* < 0.05 were considered statistically significant.

## 3. Results

### 3.1. Synthesis and Characteristics of Furano-Allocolchicinoid Derivatives

**Scheme 2:**
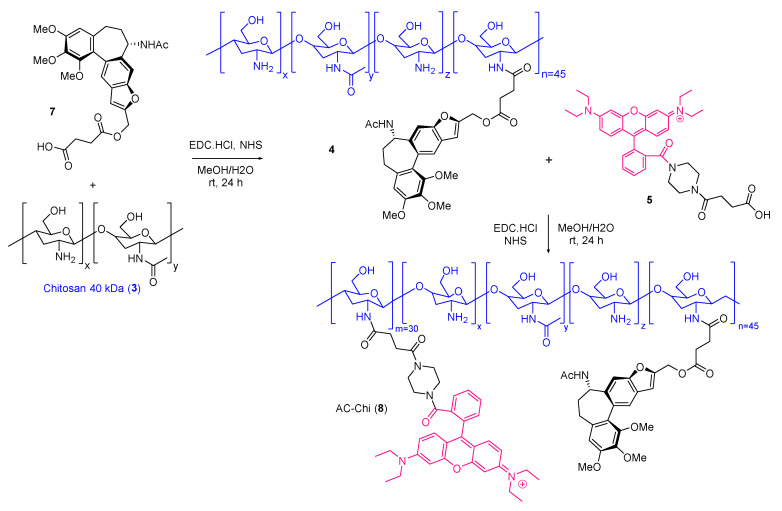
Structures and synthesis of fluorescently labeled furano-allocolchicinoid-chitosan conjugate (**AC-Chi**, **8**).

To obtain fluorescent colchicinoid **6**, or AC-chitosan conjugates **4** and **8**, furano-allocolchicinoids **2** and **7** with suitable functional groups wase synthesized as described earlier [7]. The synthesis and structures of furano-allocolchicinoid analogues are shown on Scheme 1 and Scheme 2. All conjugates were obtained using Steglich conditions; rhodamine derivative was synthesized according to the published procedure from commercial rhodamine B [26]. 

### 3.2. Nanoparticle Development and Characterization

**Figure 3:**
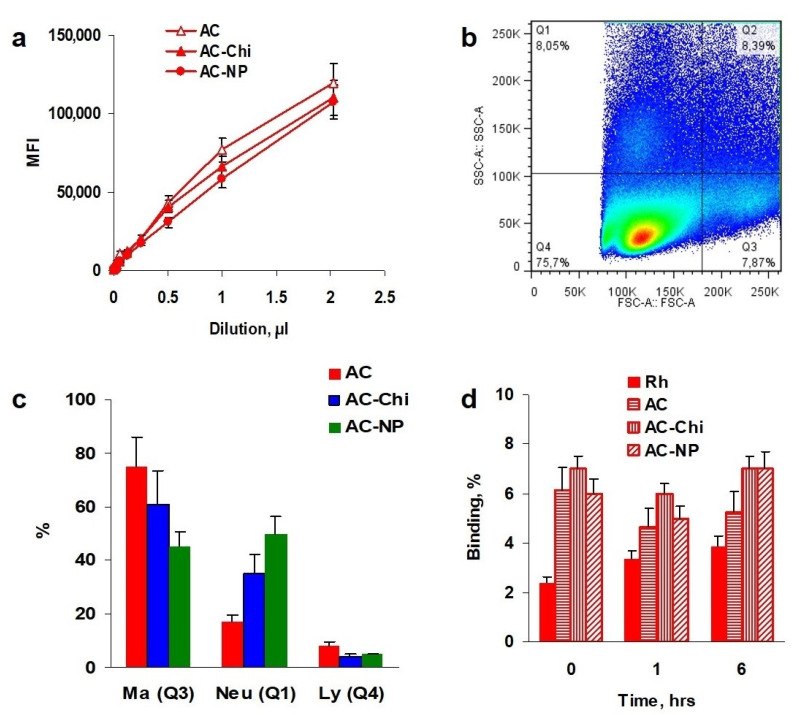
Binding of furano-allocolchicinoid **2** formulation to blood cells. (**a**,**b**) Calibration curves of rhodamine B labeled **AC**, **AC-Chi**, and **AC-NPs**. Ordinate corresponds to mean fluorescence intensity (MFI). (**b**,**c**) Furano-allocolchicinoid formulations were incubated 1 h with human whole blood and analyzed by flow cytometry. A representative dot plot of human blood cells (**a**). Lymphocytes (Ly), macrophages (Ma), and neutrophils (Neu) locate in quadrants Q4, Q3, and Q1, respectively. Percentages of Ma, Neu, and Ly after in vitro 1 h binding. (**d**) Percentage of rhodamine B and furano-allocolchicinoid formulations bound to mouse leukocytes when injected in vivo and extracted at 0.1, 1, and 6 h post injection.

NPs were developed using laurylsuccinoylchitosan derivative with deacetylation degree 95%, and substitution degree 48% and 12% for succinoyl and lauryl residues, respectively (Figure S1). **AC-NPs** were prepared by self-assembly during dialysis from organic phase to water. Functional activity of furano-allocolchicinoid in different formulations was studied by MTT assay using different human and murine cell lines (Figure 2). The anti-proliferative activity of fluorescent furano-allocolchicinoid conjugate **AC** was close to the parental furano-allocolchicinoid **2 [23]** and its IC_50_ was determined as 40 nM (Figure 2a). Estimated IC_50_ concentrations of the compound **AC-Chi** and **AC-NPs** were around 100 μM (Figure 2b). Average diameters of **AC-Chi** and **AC-NPs** estimated by DLS were 30–40 and 400–450 nm, respectively (Figure 2c,d). Zeta-potential changed from +30 mV for unmodified chitosan to +7 mV and −18 mV for **AC-Chi** and **AC-NPs**, respectively. It is likely that **AC-Chi** also forms nanoparticles due to hydrophobic properties of chitosan. **AC-NPs** additionally were visualized by confocal microscopy (Figure 2, insertion). For the in vivo and in vitro experiments all rhodamine B labeled compounds **AC**, **AC-Chi**, and **AC-NPs** were adjusted by rhodamine quantity (Figure 3a). 

### 3.3. Binding to Leukocytes

Earlier it was shown that colchicine binds to neutrophils and serum albumin [4]. Human peripheral blood leukocytes were used to analyze furano-allocolchicinoid preparation binding to leukocytes. A representative dot plot shows a gating strategy to localize neutrophils (Figure 3b, quadrant Q1), macrophages (Q3), and lymphocytes (Q4). Our results demonstrated that **AC** bound more to macrophages than to neutrophils. Conjugation of **2** with chitosan resulted in the decrease to macrophage and increase to neutrophil binding (Figure 3d). We have also estimated total leukocyte binding of AC preparations in vivo. It appeared that in 1 h experiment from 2 to 6% of tested samples were associated with blood cells (Figure 3c). It should be noted that furano-allocolchicinoid preparation bound more to blood cells than free rhodamine B. 

### 3.4. Biodistribution of AC Preparations

Analysis of substance biodistribution in animals represents quite a complex task. Several precautions were used to minimize experimental artifacts. First of all, after blood collection mice were sacrificed and residual blood from organs was removed by transcardial transfusion. Second, the same volume of homogenization buffer per a fixed mass of tissue was used to achieve an equal extraction efficacy. Third, autofluorescence levels of extracts in each type of tissue used in this work were determined and subtracted from total MFI of experimental samples. Finally, four independent experiments were conducted to have a sufficient number of mice per time point and also minimize day-to-day variability. Cooperatively 12 mice per group and time point were used. 

The yield of the extracted from the tissues conjugates with rhodamine B depends on the distribution coefficient D of the preparations and the efficiency of the extraction. To estimate a cumulative yield heparinized whole blood and homogenates of major organs were prepared. They were mixed with a fixed amount of furano-allocolchicinoid preparations and incubated 1 h at 37 °C in CO_2_ incubator. Plasma was collected from the whole blood sample and measured directly. Fluorescent species from tissue homogenate samples were extracted by the standard method and the yield was estimated. The total yield was around 30% for all types of solid organs and 60% for plasma. There were only marginal differences between the extraction efficacies of different preparations (Figure S2).

**Figure 4:**
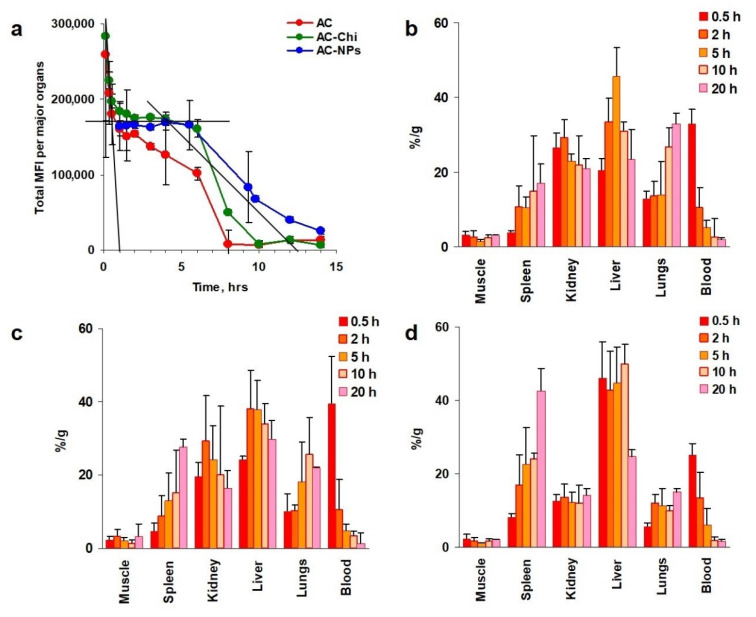
Biodistribution of furano-allocolchicinoid formulations in mice. (**a**) Total MFI collected from organs and blood serum. (**b**–**d**) **AC**, **AC-Chi**, and **AC-NPs** were IV injected and extracted at 0.5–20 h post injection. Biodistribution of **AC** (**b**), **AC-Chi** (**c**), and **AC-NPs** (**d**) in dynamics. Data are shown as percentages per gram of tissue (%/g).

Generally, the results on biodistribution are shown as percentages from initial dose injected per gram of tissue (ID%/g). This approach does not permit to identify an accumulation of the preparation due to the high excretion rate. The dynamics of excretion had two critical time points: a sharp decline during first hour, a plateau from 1 to 6 h, and slower decrease till 10 h (Figure 4a). Evidently during the first hour post injection excretory organs collect the preparations from the blood; between 1 and 6 h there is a balance between collection and excretion; after 6 h most of the preparations are already excreted. Initial dose was equal 10^6^ MFI per mouse. Consequently, the maximal yield at 30 min post injection was around 30%, close to what was obtained in in vitro experiments. Larger size preparations **AC-Chi** and **AC-NPs** were retained longer than **AC** (Figure 4a).

Figure 4b,c show a cooperative time-dependent distribution of furano-allocolchicinoid preparation between major organs. The data are shown as percentages per gram of tissue from total collected per major organs and serum at each time point (%/g). The major organ of preparation biodistribution was liver for all substances as it is shown in most papers. Amount of the preparations in other major organs such as spleen, kidney, and lungs depended on the type of the preparation. 

### 3.5. Dynamics of Biodistribution

**Figure 5:**
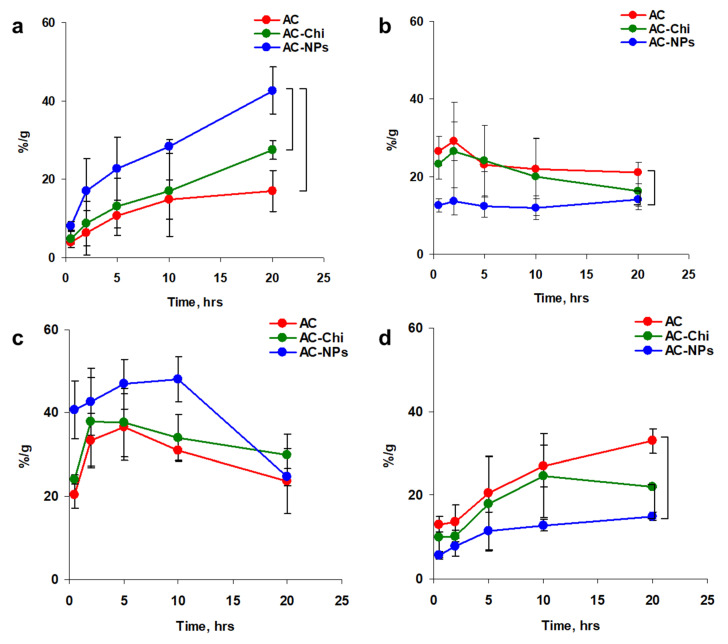
Dynamics of furano-allocolchicinoid **2** preparation accumulation in different tissues. Mice were once IV injected with a comparable quantity of formulations. Percentage of rhodamine B per gram of tissue (%/g) in liver (**a**), spleen (**b**), lungs (**c**), and kidney (**d**) at different time post injection. Significant differences (*p* < 0.05) are shown by brackets.

The dynamics of biodistribution in different organs demonstrated significant variability in all major organs between furano-allocolchicinoid preparations (Figure 5). Excretion of all preparations in liver and kidney results in a decrease in %/g with time. As expected for a larger size preparation, **AC-NPs** were found in a higher quantity (around 40–44%) and were retained in the liver up to 10 h, while the clearance of **AC-Chi** and especially of **AC** was evident starting from 6 h (Figure 5a). The second major organ for **AC-NPs** was spleen where the particles were retained significantly longer (Figure 5b). As spleen is not an excretory organ, an increase in the %/g reflects not the steady migration of nanoparticles into spleen, but retention of what was already distributed. **AC-NPs** were less accumulated and less excreted via kidney than **AC** and **AC-Chi** possibly due to their size. **AC-NPs** were also found in a lower quantity in the lungs (Figure 5d).

### 3.6. Toxicity of AC-NPs to Small Intestine

**Figure 6:**
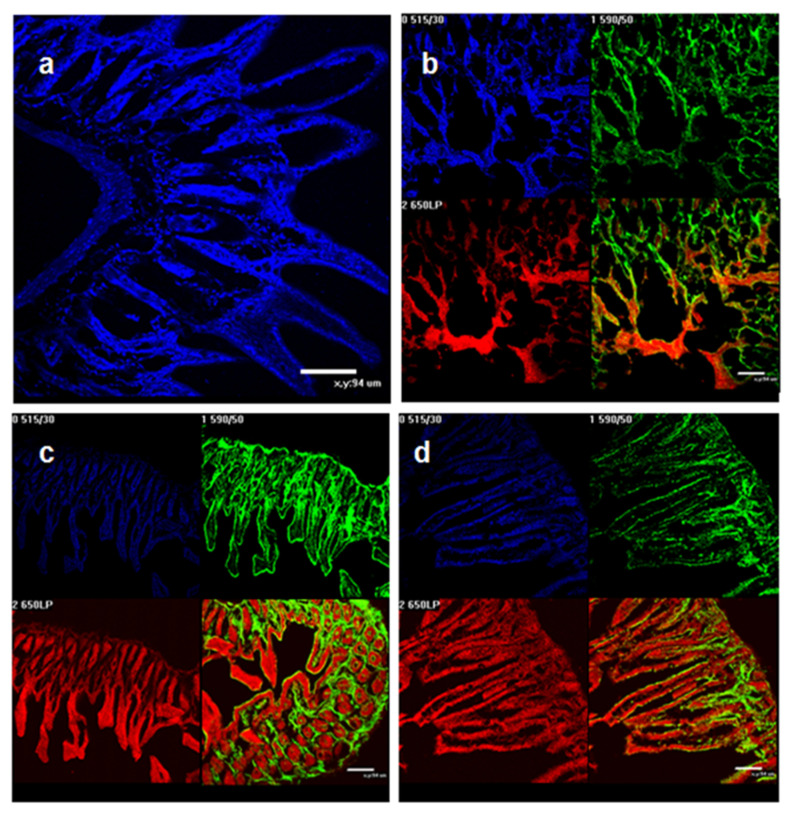
Cytohistological images of small intestine post IV injection of furano-allocolchicinoid preparations. Mice were IV injected with a comparable quantity of **AC**, **AC-Chi**, or **AC-NPs**. Small intestine was excised, washed out and cryosectioned. Sections of control intestine (**a**), **AC** (**b**), **AC-Chi** (**c**), or **AC-NPs** (**d**) 5 h post injections stained with DAPI (nuclei, blue), phalloidin (actin, green). Red color corresponds to rhodamine B in preparations. Scale bar 100 μm.

Samples of small intestine were analyzed to compare the toxicity of furano-allocolchicinoid preparation. To this end, fractions of small intestine were excised, washed from the feces, and cryosectioned. Confocal microscopy images demonstrated that both **AC-Chi** and **AC-NPs** were less toxic to intestinal villi than **AC** and preserved their structure while AC disturbed them severely (Figure 6). As the major side effects of colchicine treatment are nausea, vomiting, and diarrhea, less expressed gastrointestinal toxicity of **AC-Chi** and **AC-NPs** may help to overcome them paving a way to encapsulate colchicine derivatives application as anti-cancer therapy.

## 4. Discussion

Colchicine is an old drug with a good antimitotic activity and unique combination of properties; it has the potential to be competitive with modern drugs used in the medical practice. Recent studies show that colchicine modifications, such as its conjugation with isotope binding sites DOTA or NOTA, and radionuclide or incorporation into polymeric nano/microparticles such as mesoporous silica nanoparticles or glycopeptide dendrimers, can improve its antitumor activity [30,31,32,33]. The common features of these modifications are an increase in the size of colchicine molecule and a slower release from the drag carrier. To verify the role of a molecular size of colchicine delivery system on biodistribution and toxicity, two new imaging agents of different sizes were synthesized: **AC** with MW 1 kDa, and **AC-Chi** of 41 kDa. **AC-NPs** nanoparticles with 450 nm in diameter were developed by incorporation of **AC** into *N*-laurysuccinoylchitosan. **AC-Chi** preparation is also likely to form nanoparticles due to low solubility of chitosan at neutral pH.

Our results on biodistribution demonstrated a decreased excretion of nano/micro sized formulation via hepatobiliary root, low accumulation in kidney and lungs, and increased retention in spleen. The same longer retention of colchicine conjugates in non-excretory organs was demonstrated earlier [8,34]. However, we found an increased quantity of **AC-Chi** and **AC-NPs** in spleen only. In any case, longer retention in non-excretory organs can be translated into a better accumulation of such drugs in tumors, as has been shown by Korde et al. [34], and Erfani et al. [8]. In this work we have compared furano-allocolchicinoid preparations of three different sizes. As a result, biodistribution of medium size **AC-Chi** demonstrated an intermediate pattern between **AC** and **AC-NPs** showing a role of size of delivery system. Chitosan can also modify the effect on biodistribution. Earlier we have shown a better antitumor effect of AC-Chi which depended on the amount of AC in the preparations [7].

Decoration of toxic preparations can decrease its side effects. The major side effects of colchicine (nausea, vomiting, diarrhea) result from the block of gastrointestinal cell division by colchicine. It appeared that both **AC-Chi** and **AC-NPs** are less toxic to the intestinal cells possibly due to a partial degradation of chitosan in liver. 

Our results demonstrated that an increase in the size of the preparation results in the increase in the biodistribution. Which size of colchicine derivative carrier will provide a better effect when used as antitumor therapy should be further studied.

## 5. Conclusions

The idea of this work was to verify the hypothesis that an increase in the molecular size of colchicine can change its biodistribution and increase its accumulation in non-excretory organs. To this end two new fluorescent colchicine analogue allocolchicinoid-rhodamine (**AC**) and allocolchicinoid-rhodamine-chitosan (**AC-Chi**) were synthesized. These two molecules differed in the molecular weight at least 40 times. AC was used to develop nano/microparticles by loading AC onto laurylsuccinoylchitosan (**AC-NPs**) which additionally increased **AC** weight several times. As a result, biodistribution changed significantly in a size-dependent manner. An increase in the size of the preparation resulted in a higher quantity of the preparations in liver possibly due to a decreased excretion. At the same time, low accumulation was found in kidney and lungs, while higher in spleen. Decoration of furano-allocolchicinoid with chitosan also decreased toxicity to gastrointestinal tract. Both types of nano/micro sized formulations can be considered as promising candidates to treat hepatic tumors or inflammatory conditions such as liver fibrosis.

## Data Availability

The data presented in this study are available on request from the authors.

## Supplementary Materials

The following are available online at https://www.mdpi.com/article/10.3390/polym13132045/s1, Figure S1: ^1^H NMR spectra of chitosan 40 kDA (a) and laurylsuccinoylchitosan (b), Figure S2: In vitro extraction efficacy of AC, AC-Chi, and AC-NPs from different tissues.
