# Supplementary material for: Size-Dependent Biodistribution of Fluorescent Furano-Allocolchicinoid-Chitosan Formulations in Mice
Source: Polymers (Basel). 2021 Jun 22;13(13):2045. doi: 10.3390/polym13132045 (PMC8271848; doi:10.3390/polym13132045)
Supplement: Supplementary file 1 [file polymers-13-02045-s001.zip › polymers-1263037-supplementary.pdf]

Supplementary Information

Size-Dependent Biodistribution of Fluorescent Furano-Allocolchicinoid-Chitosan Formulations in Mice

Iuliia Gracheva [1,*], Maria Konovalova [2], Dmitrii Aronov [2], Ekaterina Moiseeva [2], Alexey Fedorov [1] and Elena Svirshchevskaya [2]

[1] Department of Organic Chemistry, Nizhni Novgorod State University, Gagarina av. 23, 603950 Nizhni Novgorod, Russian Federation; afedorovnn@yandex.ru
[2] Shemyakin-Ovchinnikov Institute of Bioorganic Chemistry RAS, Miklukho-Maklaya St. 16/10, 117997 Moscow, Russian Federation; esvir@yandex.ru
* Correspondence: yulia19gra4ova@gmail.com

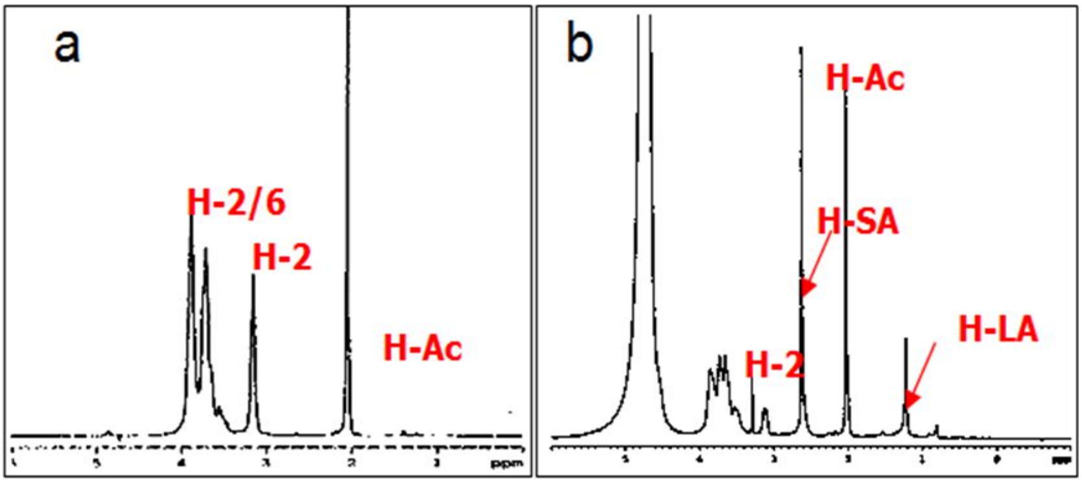

Figure S1. ^{1}H NMR of chitosan 40 kDA (**a**) and laurylsuccinoylchitosan (**b**).

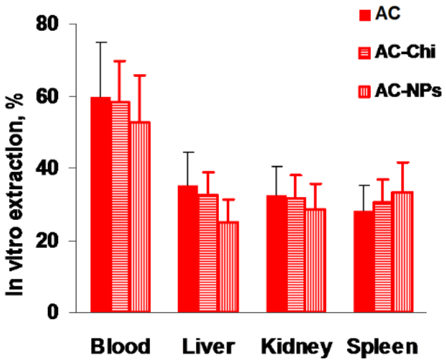

Figure S2. Extraction efficacy *in vitro* of **AC**, **AC-Chi**, and **AC-NPs** from different tissues.
